# The Healthy Start project: a randomized, controlled intervention to prevent overweight among normal weight, preschool children at high risk of future overweight

**DOI:** 10.1186/1471-2458-12-590

**Published:** 2012-08-01

**Authors:** Nanna Julie Olsen, Tine Buch-Andersen, Mina Nicole Händel, Louise Mai Østergaard, Jeanett Pedersen, Charlotte Seeger, Maria Stougaard, Maria Trærup, Kate Livemore, Erik Lykke Mortensen, Claus Holst, Berit Lilienthal Heitmann

**Affiliations:** 1 Research Unit for Dietary Studies, Institute of Preventive Medicine, Copenhagen Capital Region, Copenhagen University Hospitals, Copenhagen, Denmark; 2 Department of Environmental Health, Institute of Public Health and Center for Healthy Aging, University of Copenhagen, Copenhagen, Denmark; 3 National Institute of Public Health, University of Southern Denmark, Copenhagen, Denmark; 4 Research Unit for Dietary Studies, Institute of Preventive Medicine, Nordre Fasanvej 57, entrance 5, Frederiksberg, DK, 2000, Denmark

**Keywords:** Prevention, Obesity, Children, Susceptibility, Predisposition, Intervention

## Abstract

**Background:**

Research shows that obesity prevention has to start early. Targeting interventions towards subgroups of individuals who are predisposed, but yet normal weight, may prove more effective in preventing overweight than interventions towards unselected normal weight subsets. Finally, interventions focused on other factors than diet and activity are lacking. The objectives were to perform a randomized, controlled intervention aiming at preventing overweight in children aged 2–6 years, who are yet normal weight, but have high predisposition for future overweight, and to intervene not only by improving diet and physical activity, but also reduce stress and improve sleep quality and quantity.

**Methods/Design:**

Based on information from the Danish National Birth Registry and administrative birth forms, children were selected based on having either a high birth weight, a mother who was overweight prior to pregnancy, or a familial low socioeconomic status. Selected children (n = 5,902) were randomized into three groups; an intervention group, a shadow control group followed in registers exclusively, and a control group examined at the beginning and at the end of the intervention. Approximately 21% agreed to participate. Children who presented as overweight prior to the intervention were excluded from this study (n = 92). In the intervention group, 271 children were included, and in the control group 272 were included. Information obtained from the shadow control group is on-going, but it is estimated that 394 children will be included. The intervention took place over on average 1½ year between 2009 and 2011, and consisted of optional individual guidance in optimizing diet and physical activity habits, reducing chronic stress and stressful events and improving sleep quality and quantity. The intervention also included participation in cooking classes and play arrangements. Information on dietary intake, meal habits, physical activity, sleep habits, and overall stress level was obtained by 4–7 day questionnaire diaries and objective measurements.

**Discussion:**

If the Healthy Start project is effective in preventing excessive weight gain, it will provide valuable information on new determinants of obesity which should be considered in future interventions, and on new strategies to prevent development of overweight and obesity at an early age.

**Trial registration:**

ClinicalTrials.gov, ID NCT01583335.

## Background

The prevalence of obesity is increasing, causing a great need for developing effective intervention programs [1]. However, even if multiple controlled primary intervention studies particularly among children and adolescents have been conducted during the past 20–30 years, the majority of the studies have been unable to prevent excessive weight gain. Indeed, a first Cochrane review from 2005 showed that among studies published from 1990 through 2005, less than one in five interventions were efficient in preventing weight gain. The Cochrane review group suggested that a focus on short-term behavior change was unlikely to have a sustained impact on the weight status of children [2], and that the available evidence suggests that many diet and exercise interventions in children are in fact not effective in preventing excess weight gain, despite being effective in changing diet and exercise behavior. Similarly a recent review of interventions among preschool children aged 2–6 years did not find evidence of effective interventions [3]. Finally, a follow-up review from the Cochrane group from 2011 on intervention studies on children published from 2005 and onwards found that still only 20-30% of the published interventions were producing significant positive results. They found small, but significant intervention-control differences at the meta-level of on average 0.15 kg/m^2^ units of BMI, or 0.8% weight difference, equivalent to a 0.3 kg difference in weight for a 10 year old child. They concluded in plain language that although many studies were able to improve children’s nutrition or physical activity to some extent, only some studies were able to see an effect of the programme on children’s levels of fatness, and that combined (meta analyses) suggested that the programmes made a positive difference, but that there was much variation between the study findings which could not be explained [4].

Danish research suggests that the factors leading to the observed increase in obesity prevalence are present already in early childhood, and that prevention of obesity therefore has to start early [5]. Furthermore, it is clear that obesity is under both genetic and environmental influence, and that pre- and perinatal factors play important roles [6]. In this regard, Danielzik et al. suggests that at least three sub-groups are at high risk of becoming obese [7]:

1. *Normal weight* children with obesity among their 1st degree relatives

2. *Normal weight* children with high birth weights

3. *Normal weight* children from socially disadvantaged families (low socioeconomic status based on educational level).

Many of the previous intervention studies did not exclude children already overweight, and have consequently not been primary prevention interventions [8]. Hence, results from such interventions may not be considered as initiatives focused on primary prevention of overweight, but rather as treatment, or a mix of treatment and prevention [8].

Since a couple of recent reviews, one of which from the Cochrane group, conclude that combined behavioral lifestyle interventions compared to standard care or self-help can produce a significant and clinically meaningful reduction (1.9-3.3 kg/m^2^) in overweight in children and adolescents [9], it is likely that many previous studies that did not restrict their interventions to normal weight subjects may in fact have treated the already overweight and obese, rather than prevented the normal weight from progressing into overweight.

When it comes to primary prevention, targeting predisposed groups or individuals for interventions on preventing development of overweight and obesity, may prove to be more effective than targeting subsets of the entire population of children, because treatment rather than prevention effects are taking place, or because prevention intervention effects among the normal weight may, in reality, may be most effective among high-risk individuals, and hence be diluted and masked by general (low) effects among the non-disposed. Such scenario may provide one of the explanations for the low rate of success in previous interventions. In support of this, a recent review concluded that half of the interventions that targeted high-risk groups were in fact effective as opposed to 1 in 3–4 of the universal interventions [8].

The purpose of our intervention study “Healthy Start” [“Sund Start”] was threefold: 1) to conduct a randomized controlled intervention to prevent excessive weight gain among *normal weight* 2*–*to-6-year old children, 2) to intervene in children who were considered to be at an increased risk of future overweight and obesity, if *at least one* or more of the risk factors mentioned above was present – e.g. a high birth weight (> 4,000 g), pre-pregnancy obesity of the mother (Body Mass Index (BMI) > 28 kg/m^2^) or low social class (less than 10 years of education), and 3) to intervene with healthy sleep quality and quantity as well as stress reduction in addition to healthy eating and activity. If the intervention is effective, the results of the study will suggest that a targeted rather than universal, individual effort may be needed to prevent excessive weight gain among normal weight children.

## Methods/design

### Study design

In 2009, data on all births between 01.01.2004 and 31.12.2007 in 11 selected municipalities from the greater Copenhagen area was obtained from the Danish National Birth Registry at the National Board of Health. This register contains information on all births, whether at hospital or home, on factors such as birth weight and length, height and pre-pregnant weight of the mother, parity, and Central Personal Registry number (CPR-number). Data on socioeconomic status (SES) (estimated from maternal educational level) was obtained from the administrative birth forms. This was done manually using the CPR-numbers obtained from the birth register. This showed to require more resources than expected, and it was consequently chosen to select children from low SES families in just one municipality. After selecting the children eligible for participation, the children were allocated to three groups, using computer based randomization stratified on municipality and with simple randomization within strata, and were afterwards identified and invited to participate in the study. All siblings were allocated to the same group.

The three groups were:

#### The intervention group

Participants in this group were invited to see a health consultant trained in dietetics and nutrition. At the first meeting, heights and weights were measured and BMI was calculated. International cut-offs for overweight according to age and gender, developed by Cole et al. were applied [10]. If a child was found to be overweight it was excluded from the intervention study after this first consultation. If a child was normal weight it was included, and had additional anthropometric measurements taken. Each participating family was assigned to the same health consultant, who followed the family throughout the project period, in order to ensure confidence and personal contact. Each child and its family were seen on a regular basis, and it was possible to have up to 10 visits over a 1½-year period, where consultation frequency was based on needs and resources of the individual families. A more thorough description of the intervention and measurements obtained from this group will be presented below.

#### The control group

Children in this group were invited to see a health consultant. At the first meeting their heights and weights were measured and BMI was calculated. The same international cut-offs for overweight according to age and gender as in the intervention group were applied to the control group [10]. If a child was overweight at baseline it was excluded from the control group, and did not participate further. If a child was normal weight it was included, and hadadditional anthropometric measurements taken.

Children from the control group and their families were invited to a follow-up approximately 1½ year after the first visit, but they were not seen between baseline and follow-up.

All families that were invited to see the health consultant prior to intervention (intervention and control group) were told that their child had an increased risk of future obesity. This information may influence future health attitudes and practices, and may be considered an intervention by itself. We therefore applied a design using two control groups rather than only one, because differences in weight development between the intervention group and the control group, and hence development of overweight, could potentially be diluted as a consequence of informing the control participants that the child was predisposed to obesity.

An additional control group (the shadow control group) was therefore chosen, where the families were unaware of whether their child was predisposed or not. Children in this group were not invited to see a health consultant, and were followed exclusively through registers. Information on height and weight was obtained from records of the general practitioners (GPs) who perform regular preventive child health examinations of pre-school children at ages 2, 3, 4, and 5 years. If a child was overweight at time of baseline, it was excluded from the study. Collection of data on height and weight from the GPs is currently on-going. However, as participation in the shadow control group is unaffected by willingness to participate (information for everyone included in this group is obtained via existing registries), differences in weight development between the intervention group and this control group and hence development of overweight, will potentially be inflated since these families do not have to actively show up to become a participant. Thus, it is likely, that the true effects of the intervention may be in the range between the effects of the intervention group compared to the shadow control group, and the intervention group compared to the control group. This use of the study design may, on the other hand, provide an additional result related to whether the simple information given to the families that their child is predisposed to future overweight, may lead to self-selected changes in lifestyle without further help (as described above), and may thus prevent the development of overweight. Hence, differences between the shadow control group and the ordinary control group in excess weight gain will also be examined.

The study is registered with ClinicalTrials.gov, ID NCT01583335.

### Enrolment

Based on the information obtained from the Danish National Birth Register and the administrative birth forms, children having either a high birth weight (≥ 4,000 gr.), a mother that was overweight prior to pregnancy (BMI ≥ 28 kg/m^2^), or a mother with ≤ 10 years of schooling were selected. A BMI ≥ 28 kg/m^2^ was applied as cut-off for maternal overweight, since BMI ≥ 30 kg/m^2^ would have led to too few eligible children in the municipalities, and BMI ≥ 25 kg/m^2^ was considered too low to reflect a real biological pre-disposition to overweight in the child.

After the selection and randomization, each child was checked in the Central Personal Registry, which contains a systematic registration on all Danish citizens regarding names, address, civil status, place of birth registration, and other basic information. Children were excluded from the study if they had moved to another municipality after they were born, if they were protected from being contacted by researchers^a^, if they did not have a permanent address, lived in a children’s home, had died, had emigrated, temporally lived abroad, or had disappeared^b^. In total 5,902 children were selected. Of these 5,902 children, 2,180 (37%) were excluded from being contacted and potentially enrolled because of one or more of the above mentioned exclusion criteria. During the study’s enrolment phase, the aim was to enroll the oldest children first. However, this led to 365 children being excluded from invitation to participate due to other causes than previously described, such as having had an older sibling invited previously.

In total 3,722 children met the inclusion criteria, and were invited to participate in the study.

**Figure 1:**
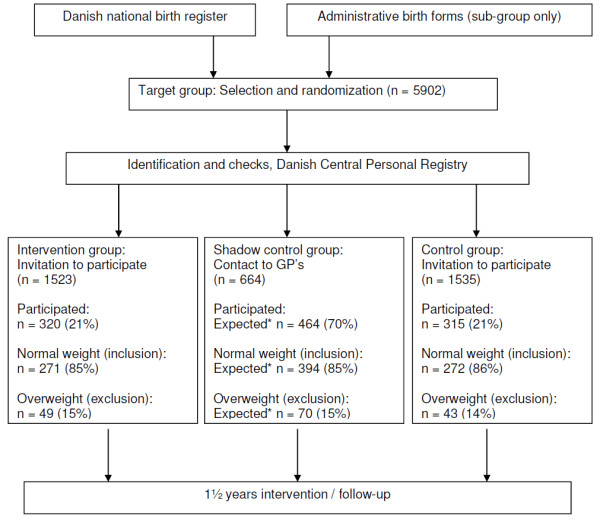
**Flow diagram of the Healthy Start project.** * = n is the expected number of participants from which information on weight development will be obtained from GP’s, and is hence not the final number of participants in control group 1.

Figure 1 shows the number of participants selected and invited to participate in the Healthy Start project. When planning the project, it was anticipated that about 40% would accept the invitation to participate. However, as shown in Figure 1, the participation rate for the intervention group and the control group was around 21%. The relatively low participation rate may reflect the focus of primary prevention, since it may be difficult to encourage lifestyle changes to parents of a child who is still normal weight, although at increased risk of developing overweight. Furthermore, it was also estimated that a maximum 10% of the participating children would be overweight, but as much as 15% of the children who turned up for the initial examination were already overweight at baseline (Figure 1). Again, this higher proportion may reflect that parents to the overweight children were more willing to show up, because they were aware that their child was at risk. Table 1 shows the baseline distribution of gender, selection on birth weight, selection on maternal pre-pregnancy overweight and selection on low SES.

**Table 1:** Pre-intervention characteristics by group

	**Intervention group (n (%))**	Shadow control group* (n (%))	**Control group (n (%))**
**Boys**	150 (55.4)	376 (56.6)	166 (61.0)
**Girls**	121 (44.6)	288 (43.4)	106 (39.0)
**High birth weight** *** only***	163 (60.2)	384 (57.8)	173 (63.6)
**Maternal pre-pregnancy overweight** *** only***	74 (27.3)	152 (22.9)	68 (25)
**Low socioeconomic status *only***	8 (3.0)	74 (11.1)	8 (2.9)
**High birth weight** *** and *** **maternal pre-pregnancy overweight**	24 (8.9)	46 (6.9)	17 (6.3)
**High birth weight *and* low socioeconomic status**	1 (0.4)	6 (0.9)	4 (1.5)
**Maternal pre-pregnancy over weight** *** and *** **low socioeconomic status**	1 (0.4)	0 (0.0)	1 (0.4)
**High birth weight *and *** **maternal pre-pregnancy over weight *and *** **low socioeconomic status**	0 (0.0)	2 (0.3)	1 (0.4)

* = n is the number of participants from which information on weight development is obtained from GP’s, and is hence not the final number of participants in control group 1.

### Measures

From the shadow control group, information on heights and weights was collected from the journals of the GP’s (on-going July 2012). As part of the child health examinations and vaccination programs, the GP measures heights and weights at age 2, 3, 4, and 5. The examinations are free of charge, and more than 80% of all Danish children participate at each examination [11].

In the intervention and the control group, information on the following variables was obtained both before and after the intervention:

#### Anthropometric measurements

Height to the nearest 0.1 cm. was measured using stature meter (Soehnle 5,002 or Charter ch200P). The children were in bare feet or in stockings. Body weight was measured to the nearest 0.1 kg using a mechanical weight or beam-scale type weight (TanitaBWB-800 or SV-SECA 710). The children were measured in underwear only, and were asked to urinate before the weighing. If the child was using diaper, a new diaper was put on before the weighing. Waist circumference was measured to the nearest 0.5 cm midway between the lowest rib and the iliac crest. Hip circumference was measured to the nearest 0.5 cm, at the place where the circumference was the largest, seen from the frontal and medial angles. Both waist and hip circumferences were measured in triplicate and a mean was calculated. Skinfolds were measured at biceps, triceps, subscapular and suprailiac on the left side of the child, using Harpenden Skinfold Caliper or Lange Skinfold Caliper. The caliper was placed perpendicularly to the skinfold right under the fingers of the person taking the measurements. After 1–2 s the caliper was read to the nearest 1 mm. Each measurement was taken in triplicate. Bioelectrical impedance was measured to estimate the child’s percentage of body fat at resistance 50 kHz (SEAC Multiple Frequency Bio impedance Meter (model SFB3 and SFB2 version 1.0), RJL or Animeter (BIA-101 and BIA-103)). Prior to the measurements, the child was asked to lie quietly in a supine position with the legs and arms slightly apart. An alcohol wipe was used to remove excess skin oils to secure attachment of the electrodes. Measurements were taken on hand and foot of the child’s right side. On the hand, the first electrode was placed right below the joint of the middle finger and the second electrode right midway between the two bones on the dorsal side of the wrist. On the foot, the first electrode was placed below the joint of the 3^rd^ toe and the second electrode right between the two large bones in the ankle on the dorsal side of the foot. Bioelectrical impedance measurements were taken twice and a mean was calculated.

#### Dietary measurements

The parents of each participant were asked to fill out a 4-day dietary record of their child. The parents were asked to record the dietary intake from Wednesday to Saturday. These specific days were chosen to obtain information on dietary intake in both week- and weekend days. The dietary record was accompanied by a picture book to help the families estimate portion sizes and a guidance in how to fill out the dietary record. Each dietary record was entered into Dankost 3000, a software program applied to calculate the individual intake of macro- and micronutrients.

#### Parental questionnaire

A questionnaire completed by the parents provided information on meal habits, physical activity habits, sleep habits, stress in the child, overall well-being of the family, general questions about the family and its structure and relationships, and paternal and maternal basic demographic and social information (height, weight, educational level, occupation, amount of work hours and hours spend outside the home without the child).

#### Meal habits

The questions on meal habits concerned the child’s appetite and way of eating (picky or not), number of times per week the family had breakfast or dinner together, if the family meals were considered to be cozy or conflict-ridden, priorities about serving vegetables or fruit, and who served vegetables on the child’s plate.

#### Physical activity

Information on physical activity was obtained by including the 7-day Children’s Physical Activity Questionnaire (C-PAQ) as an integrated part of the parental questionnaire [12]. The C-PAQ questionnaire was modified in order to make the activities match with the possibilities for physical activity for Danish children aged 2–6 years. Questions were also asked on the distance between home and day-care, means of transportation to and from day-care, the number of hours spend outdoor during day-care, the estimated level of physical activity in the child compared to other children of similar age, frequency of which one or both parents were physically active with the child, and if the child enjoyed being physically active.

#### Sleeping habits

Parents reported sleep habits in a 7-day sleep diary and also answered questions related to whether or not the child had problems falling asleep in the evening, problems waking up in the morning, if it was difficult putting the child to sleep, sleep onset latency, if the child took naps during the day (and if so, for how long time), routines with the child before bedtime, if the child entered the parents’ bed during night (and if so, how often), if the child was afraid when trying to fall asleep, if the child had nightmares, if the child was sleeping calmly, and if the child had difficulties falling asleep again after waking up during the night. Thus, the questions provided information on sleep duration and markers of sleep quality.

#### Stress of the child

To assess behavioral difficulties and stress in the child, the Danish version of the Strengths and Difficulties Questionnaire (SDQ) was included as an integrated part of the parental questionnaire. SDQ is a behavioral screening questionnaire that consists of 25 items on prosocial behavior and emotional and behavior problems. Scores on 5 scales are derived from these 25 items: emotional symptoms (5 items), conduct problems (5 items), hyperactivity/inattention (5 items), peer relationship problems (5 items) and pro-social behavior (5 items) [13]. The SDQ has been translated into the different Nordic languages, and has so far been completed for nearly 100,000 children in the Nordic countries during the past years [14].

Table 2 shows the pre-intervention distribution of age, height, weight, BMI, waist circumference, hip circumference, sum of 4 skinfolds, percentage body fat, energy intake, carbohydrate intake, protein intake, fat intake, sugar intake, sleep latency, SDQ-score, and household income by group. *T*-test analyses showed a statistically significant difference in total SDQ-score between the intervention group and the control group (Table 2). No other significant differences in baseline characteristics between the intervention group and the control group were found (Table 2).

**Table 2:** Pre-intervention distribution of selected covariates by group

	**Intervention group**			**Control group**			**P (*t*-test)**
	**n**	**Mean**	**SD**	**n**	**Mean**	**SD**	
**Age (years)**	271	4.0	1.1	272	4.0	1.1	1.00
**Height (cm)**	271	104.3	9.2	272	104.5	9.1	0.77
**Weight (kg)**	271	17.4	3.1	272	17.6	3.0	0.45
**BMI (kg/m**^**2**^**)**	271	15.9	1.0	272	16.0	1.0	0.12
**Waist circumference (cm)**	253	51.8	3.2	257	52.1	3.0	0.22
**Hip circumference (cm)**	252	55.5	3.6	255	56.0	3.9	0.19
**Sum of 4 skinfolds (mm)**	236	24.6	5.4	242	24.8	5.2	0.67
**Body fat (%)**	199	22.3	10.1	176	21.3	8.9	0.33
**Energy intake (MJ)**	237	4.7	1.0	244	4.8	1.0	0.15
**Carbohydrate intake (gr./day)**	237	158.0	36.0	244	164.1	35.3	0.06
**Protein intake (gr./day)**	237	42.8	11.2	244	44.6	10.3	0.07
**Fat intake (gr./day)**	237	38.1	10.1	244	38.1	11.2	0.96
**Sugar intake (gr./day)**	237	56.2	16.5	244	58.5	16.5	0.12
**Sleep latency (minutes)**	250	18.3	14.5	256	18.6	13.9	0.84
**SDQ-score (points)**	253	7.1	3.9	256	6.4	4.0	0.05*
**Household income (thousands, DKK)**	217	870	622	231	788	306	0.07

#### Overall well-being of the family

The Parental Stress Index (PSI) is a self-reported inventory designed to measure parental experiences of stress in relation to the parent–child interaction. It has six child subscales and seven parent subscales. In the Healthy Start project, a modified Swedish version [15] was included, as an elaboration of the parental part of the questionnaire. The questions included information on perceived changes in life since the child was born with regard to factors such as sleep, work, stress, life satisfaction, conflicts with spouse, and worries. Moreover, one question asked if the parent thought he/she had enough time together with the child.

#### General questions about the family and its relations

This section of the parental questionnaire included information on paternal, maternal, and child ethnicity, whether the parents were living together, number of siblings, household income, chronic physical diseases in the child, mental diseases in the child, and estimated general physical health of the child.

#### Paternal and maternal basic information

Both the mother and father were asked if he or she was the biological parent of the child, and about their heights and weights, their highest completed educational level, occupation, average number of work hours per week, average number of hours per week spend outside the home without the child per week, as well as their level of physical activity per week.

#### Biological samples

During the last or second to last visits we sampled hair and saliva from children and their parents in the intervention- and the control group.

Hair was sampled from each child and both parents to obtain an objective measure of chronic stress. Long-term cortisol can be used as an indicator of both sub-acute and chronic stress, as analysis of cortisol in hair potentially gives a non-invasive measurement of long-term activity in the HPA-axis [16]. It has previously been found that hair from human individuals may be used as an integral measure of cortisol production in the past 3–6 months [17]. As hair grows approximately 1 cm per month, it is estimated that the first cm of hair (from the scalp) contains cortisol from the previous month, whereas the next one cm of hair contains cortisol from two months back and so on [17]. Information on frequency of hair washes and whether the hair was currently colored was also obtained, in order to adjust for potential dilution of the cortisol level. Compared to subjective measurements of stress derived from inventories, the hair analyses may provide a more accurate measure of chronic stress level, by estimating cortisol over the previous 1–6 months. The measure may hence be used to study how chronic stress may affect the body [16], and in particular the risk of developing overweight and obesity. Cortisol will be extracted from hair using the Elisa method [18]. The biochemical extraction of cortisol is still on-going, but is expected to be ready fall of 2012.

#### Saliva

Saliva samples for each child and both parents were also collected, using Oragene OG-500 and OG-575. OG-500 was used for the parents and children who were able to spit on their own. They were asked to spit 2 ml in a tube, which was afterwards closed and shaken for 5s.

OG-575 was used for children who were not able to spit on their own. A swab was placed and moved in cheek pouch along the gums and inner cheeks to absorb as much saliva as possible. The swab was then placed in a v-notch of funnel, and saliva was wringed out of v-notch into the tube. The tube was then closed and mixed for 5 s. DNA is currently being extracted from the samples and specific SNP analyses or GWAS will be performed later in relation to candidate genes related to obesity and weight gain, food dependency, appetite, hunger and satiety, physical activity and sedentariness, stress coping or sleep quality (melatonin and clock genes).

#### Actigraph GT3X

As a means of validating the questionnaire information, objective information on daily activity as well as sleep duration and sleep quality was obtained on a subgroup of the intervention children over 5 days/nights (n = 79) half way through the intervention period, using Actigraph GT3X. Actigraph catches a person’s movements triaxial through an accelerometer on one single axis or multiple axes. The device catches movements between 0.25 Hz – 2.3 Hz, as previous studies have found that voluntary movements take place within this range [19]. Specifically, information on sleep efficiency (sleep/number of hours spend in bed), time the child went to bed, time the child got out of bed, time asleep, awakenings, sleep onset, minutes awake, average duration of awakening and sleep latency, was obtained. The child was asked to wear the Actigraph for 5 successive days, and the parents were instructed that if the child did not want to wear the device all the time, the most important time to wear it was during the night. Moreover, parents were asked to complete a sleep diary for the same days that the Actigraph was worn. The device was placed on the left wrist (right wrist if the child was left-handed) using an elastic band. If the elastic was too big for the wrist, it was placed on the upper arm or the ankle (left ankle if the child was right-handed and vice versa). Actigraph GT3X was also used for obtaining objective information on physical activity over five consecutive days (n = 79). The device was set to an epoch length of 60 s with normal filter level.

Finally, from all included children (intervention- and both control groups), information and measures of various factors during the child’s first years of life is available from a clinical database which visiting nurses from 12 municipalities from the Copenhagen area have established. This clinical database includes standardized records with information on breast-feeding, exposure to passive smoking, maternal health, decreased ability to care for the child because of alcohol or other substance abuse, network in the family, remarks about the child’s signals and reactions (sleep, anxiety, crying), and growth during the child’s first years of life. Information from this database will be obtained by linkage with the CPR-numbers.

### Power calculation

With a power of 90%, alpha of 0.05 and 275 individuals in each group, it will be possible to detect a difference between the intervention- and control groups of 0.28 SD in BMI. For a 7-year old boy with a height of 1.25 m (equivalent to the mean height for 7-year old children in 1980–1983), a 0.28 SD BMI corresponds to a difference in BMI = 0.45 kg/m^2^, or 0.70 kg body weight between the intervention and control groups. With 80% power and a significance level of 5%, we will be able to demonstrate differences of 0.5 BMI units between the intervention and control group children. We greatly oversampled in relation to the main outcome (BMI) to secure sufficient numbers post-intervention and in relation to more specific outcomes such as body fat and fat free mass.

### Intervention

The intervention consisted of four main areas: optimizing dietary intake and physical activity quantity and quality, improving sleep duration and quality, and reducing chronic stress of the child and the family by improving interaction between child and parents. The intervention was planned and organized according to each participating family’s individual needs and resources. Thus, the intervention was not administered as a standardized package that was more or less identical for all participants. Based on primarily the dietary record and the parental questionnaire, the consultants evaluated the specific needs of each family, and subsequent consultations were planned to include one or more of the four themes (ex. intake of sugar, intake of vegetables, physical activity outside day-care, bed time routines etc.). This evaluation was also based on the themes each family was motivated to work with, using the “stages of change” motivational models. Translational assistance was not provided by the project, and consequently non-Danish speaking individuals were not able to participate.

The main focus of the intervention was on the family as a whole and not only on the child. Stages of change principles and motivational interviewing were used as the framework for the counseling process [20], and the main concept was to improve the family’s knowledge and action. Motivational interviewing (MI) is a psychotherapeutic method that is evidence-based and is applicable across a wide variety of problem areas including health behavior change [21]. MI uses a guiding style to engage with people, clarify their strengths and aspirations, evoke their own motivations for change, and promote autonomy of decision making [22], and was applied to enhance personal motivation for change. In the stages of change model, change is a process involving progress through a series of stages. MI was used to help families to proceed through these stages [23].

Each consultation started with asking the family if they had any themes they wanted to discuss, and how problems or agreements had progressed since the previous consultation. In addition to the consultations, families in the intervention group were invited to participate in bi-monthly cooking classes and monthly play (activity) classes. The idea behind these classes was to inspire the participants to convert the theory from the consultations into practice, to motivate the family to spend more time together and to network with other families. The cooking classes had various themes, such as using fish in the meals, healthy fast food, healthy desserts etc.

Keywords for each of the four main intervention areas were prepared to support the health consultants in their planning of the consultations. For these keywords, tools to change behavior in a given direction were created, and together with the consultant each family then decided which tools they wanted to work with until next consultation. Examples of tools are presented in Table 3.

**Table 3:** Examples of tools compi led from the key words selected within each intervention focus area

	**Diet**	**Physical activity**	**Sleep**	**Stress**
**Example of tools**	· Make fruit a part of breakfast or dessert in the lunch package	· Plan activities with the whole family together	· Avoid that the child uses computer games, internet or watches TV before bedtime	· Prepare the child of what will happen during the day
	· Supply the lunch · package with small tomatoes, carrot sticks etc.	· Go with the bike, for a walk or play outside with your child	· Avoid that the child watches TV-programs catered for adults	· Build up every-day routines
	· Replace the sugary cereals with oatmeal	· Restrict screen time to 2 hours per day	· Increase the physical activity level of the child during daytime	· Distinguish between work- and free time, so the child knows when it has the parent’s full attention
	· Eat natural yoghurt and add with fruits of your own choice		· Provide clear rules and routines around bedtime	· Be physically active with your child every day
	· Restrict intake of sweets, but when provided, use for instance fruit sorbet as an alternative to ice cream, fruit as an alternative to sweets etc.		· Avoid a stressed environment in the home	
	· Keep a jug of water in the refrigerator, and refresh the water with lemon slices, fresh mint leaves or frozen fruit			

The keywords for the four main areas were:

#### Dietary intake

The chosen keywords were based on the Danish national recommendations, “The 8 Dietary Advices”. Each of the 8 dietary advices were included, but adjusted to the age of the target group (2–6 years). Thus, examples of the key points were:

· Eat fruit and vegetables, 300–500 grams per day, depending on the age

· Eat fish or fish filling several times per week

· Eat potatoes, rice or pasta and whole grain bread every day

· Go easy on sugar, especially from soft drinks, sweets and cake

· Go easy on fat, especially from dairy products and meat

· Eat from all food categories every day, choose various products within each food category

· Quench your thirst with water, 1-1½ liter per day

· Be physically active, at least 1 hour per day [24]

#### Physical activity

Based on current literature, examples of the selected key points were:

· Increase parental physical activity and interaction. Children who are physically active with their parents are more active than children who are not [25]

· Increase time spend outdoor. Children who spend more time in outdoor play spaces are more active than children who spend less time outdoor [25]

· Reduce sedentary behavior. TV viewing and sedentary behavior may be associated with physical activity in children [25]

#### Sleep

Based on current literature, the selected keywords were:

· Reduce time spend on computer games, internet and both active and passive TV-watching before bedtime [26,27]

· Increase daytime physical activity level [28]

· Improve the sleep environment in the home. It seems that clear rules and routines around bedtimes improve children’s sleep quality, whereas the presence of parents while the child is falling asleep has the opposite effect [29]

· Improve the emotional environment in the home. Parental stress has been shown to be associated with poor sleep quality [30]

#### Stress

A literature search showed that social isolation, attendance in full-day pre-school [31] and major life events [32] were important determinants of psychological stress in the child. Moreover, a high level of parental stress was linked to insecure child attachment and parental unresponsiveness, both of which are known to induce psychological stress in the child [33]. Thus, some of the key points in this part of the intervention were:

· Increased parental awareness of the stress-level

· Attention to being present when being with the child

· Increased physical activity

· Tranquility to quiet playing

· Physical contact, e.g. plays, sports and massage [34]

As it emerges from Table 3, the various parts of the intervention were often integrated – i.e. focusing on the area of physical activity may additionally benefit sleep quality and reduce the stress level of the child.

### The team

The daily project team consisted of a research director, a project leader and three health consultants. Trainees and student workers were connected to the project continuously. Moreover, the project had supervision from statisticians, psychologists and dieticians on designing and carrying out the study.

### Data cleaning

Data was entered into the Epidata system and subsequently cleaned.

Baseline data was cleaned first by going through all questionnaires, dietary records, measurements and notes from the consultations, and comparing information from the paper forms with information in the database. This comparison was conducted for six variables, which were selected based on a high proportion of missing information. Then, information from questionnaires, dietary records and measurements from 5% of the participants was re-entered, and it was decided to accept an error rate of 2%. The same procedures will be performed with follow-up data.

### Ethics

The Danish Data Protection Agency approved of the study (journal number: 2007-41-0530). The Scientific Ethical Committee of the Capital Region in Denmark decided that according to Danish law, the project should not be submitted to the committee (journal number H-A-2007-0019). Informed consent to use the collected data for research purpose was obtained from all participant’s parents.

### Future follow-up

Future weight development will be followed for participating children in the intervention and both control groups by first linking the CPR-numbers to the school-health records (heights and weightsare measured at enrolment (age 6–8 years), midway (age 10–12 years), and at termination (age 14–16 years)), then to draft board records for the males (draft board examinations are mandatory in Denmark, and includes, among others measurement of heightsand weights) and to the Danish National Birth Registrywhere information on weights and heights can be obtained, because pre-pregnancy weights and heights is recorded during the first 6–10 weeks of all pregnancies. Thus, the project has important potential for evaluating the long-term effects of the intervention.

## Discussion

This primary intervention study described in this paper aims to prevent development of overweight among those children aged 2–6 years, who are still normal weight but also are at high risk of future overweight, because of either a high birth weight, having a mother who was overweight prior to pregnancy, or having a mother with low educational level. The study is considered to be the first to perform an intervention focusing on young high-risk children. Additionally, it is also among the first studies to intervene not only by improving diet and physical activity habits, but also by optimizing sleep quality and quantity, and reducing stress levels. Finally, few prior studies considered normal weight children only, but included both normal and overweight children in their interventions.

Our study will be able to assess not only the short-, but also the long-term effects of an early intervention, due to the possibility to link the participants’ CPR-numbers to relevant Danish registers, with information on weight development during school years and way into adolescence and adulthood.

The limitations of the study include attrition bias, since it is likely that the most resourceful families will remain in study. This will be explored by describing attrition according to maternal BMI and SES, and the child’s birth weight which are known for both participants and non-participants. Randomization should eliminate the problem with a lower than expected participation rate. However, it may inflate observed differences between intervention and control group since the participating families may be those who already focus on healthy lifestyles, and who are the most resourceful.

The present study focused on the overweight mothers, and did not consider overweight among fathers. Generally, predisposition to obesity in a child is dependent of whether the mother or the father is overweight or obese, but unfortunately no Danish registers contain information on paternal heights and weights around the time the child was conceived or born, which could have been used for selection of the children predisposed to future overweight in the present study. If the result of the intervention is found to be effective in future, information on familial obesity may be obtained from the public health nurses who have contact with almost all families from birth through 1 year of the child, or through GP’s who generally have contact to families on a regular basis including during the child’s first years of life. Also, the mandatory draft board examinations may be used to identify those fathers who were overweight already at age 19 on average. Finally, school health registries can be used to identify children with overweight siblings.

An important strength of the study is the randomized design and the use of two control groups, which increases the possibility to estimate a true effect of the intervention. This design also makes it possible to evaluate if the simple information to parents that their child is at a high risk of future overweight, is an easy and inexpensive way of preventing some later overweight in the child. Another important strength is the use of hair samples to measure long term stress exposure via cortisol level from around 350 children and 650 parents, which is considered to be one of the largest sample of hair cortisol collected till date.

Indeed, if the intervention proves to be effective, it may easily be integrated into the already established and very efficient Danish visiting nurses system, where families are offered home visits from a nurse and counseling on ex. the child’s well-being, lactation, and development during the child’s first two years of life. It may also be an independent program in municipalities who wish to draw special attention to pre-school children and/or primary prevention of overweight and obesity.

The Danish Board of Technology recently published a report that focused on action on targeted prevention of obesity. It was concluded that “in order to curb the obesity epidemic, effective ….. prevention in yet *normal* weight is needed”. It was summarized that there is a need for improving the effect of obesity prevention in those groups who are at the *highest future risk* of developing overweight and obesity [35], and that future efforts therefore should *target high risk groups* to a higher extent [35]. It is considered that the Healthy Start project is in line with this conclusion, and thus may provide the first important information on the effectiveness of such programs, and if effective, in addition contribute with evidence on how to curb the obesity curve.

## Conclusion

In conclusion, the Healthy Start project aims at preventing overweight among pre-school children who are still normal weight, but at risk of future overweight. If the study is effective, it may provide valuable information on new determinants of overweight and obesity and a new strategy to prevent development of overweight and obesity at an early age.

## Endnotes

^a^Protection from being contacted by researchers provides protection from participation in statistical and scientific surveys, based on data delivered from the Danish Central Person Registry, From 2000 to 2007 protection from being contacted by researchers was linked to moving, as it was obtained by ticking off a box when filling out moving certificates. Since 2007 it was removed from the moving certificates, and replaced by a separate certificate that needs to be filled out.

^b^A person is considered to be disappeared if an individual has been searched for as being disappeared or if its life status is unknown.

## Competing interests

All authors declare no competing interests.

## Authors’ contribution

NJO conceived the study, was responsible for its design and coordination, participated in the data collection and drafted the manuscript. TBA carried out the data collection and helped to draft the manuscript. MNH participated in the data collection and helped to draft the manuscript. LMØ carried out the data collection and helped to draft the manuscript. JP participated in the data collection and helped to draft the manuscript. CS carried out the data collection and helped to draft the manuscript. MS participated in the data collection and helped to draft the manuscript. MT carried out the data collection and helped to draft the manuscript. KL helped to draft the manuscript. ELM participated in the design of the study and helped to draft the manuscript. CH was responsible for the randomization and statistical support and helped to draft the manuscript. BLH conceived the study, was responsible for its design and coordination and helped to draft the manuscript. All authors read and approved the final manuscript.

## Pre-publication history

The pre-publication history for this paper can be accessed here:

http://www.biomedcentral.com/1471-2458/12/590/prepub
